# Autoantibodies to Vasoregulative G-Protein-Coupled Receptors Correlate with Symptom Severity, Autonomic Dysfunction and Disability in Myalgic Encephalomyelitis/Chronic Fatigue Syndrome

**DOI:** 10.3390/jcm10163675

**Published:** 2021-08-19

**Authors:** Helma Freitag, Marvin Szklarski, Sebastian Lorenz, Franziska Sotzny, Sandra Bauer, Aurélie Philippe, Claudia Kedor, Patricia Grabowski, Tanja Lange, Gabriela Riemekasten, Harald Heidecke, Carmen Scheibenbogen

**Affiliations:** 1 Institute of Medical Immunology, Charité—Universitätsmedizin Berlin, 13353 Berlin, Germany; marvin.szklarski@charite.de (M.S.); sebastian.lorenz@charite.de (S.L.); franziska.sotzny@charite.de (F.S.); sandra.bauer@charite.de (S.B.); claudia.kedor@charite.de (C.K.); patricia.grabowski@charite.de (P.G.); carmen.scheibenbogen@charite.de (C.S.); 2 Department of Nephrology and Critical Care Medicine, Charité—Universitätsmedizin Berlin, 13353 Berlin, Germany; aurelie.philippe@charite.de; 3 Department of Rheumatology and Clinical Immunology, University of Lübeck, 23538 Lübeck, Germany; tanja.lange@uksh.de (T.L.); Gabriela.Riemekasten@uksh.de (G.R.); 4 CellTrend GmbH, 14943 Luckenwalde, Germany; heidecke@celltrend.de; 5 Berlin Institute of Health Center for Regenerative Therapies (BCRT), Charité—Universitätsmedizin Berlin, 10117 Berlin, Germany

**Keywords:** adrenergic receptors, autoantibodies, myalgic encephalomyelitis, chronic fatigue syndrome, autoimmunity, vasoregulation, G-protein-coupled receptor

## Abstract

Background: Myalgic Encephalomyelitis/Chronic Fatigue Syndrome (ME/CFS) is an acquired complex disease with patients suffering from the cardinal symptoms of fatigue, post-exertional malaise (PEM), cognitive impairment, pain and autonomous dysfunction. ME/CFS is triggered by an infection in the majority of patients. Initial evidence for a potential role of natural regulatory autoantibodies (AAB) to beta-adrenergic (AdR) and muscarinic acetylcholine receptors (M-AChR) in ME/CFS patients comes from a few studies. Methods: Here, we analyzed the correlations of symptom severity with levels of AAB to vasoregulative AdR, AChR and Endothelin-1 type A and B (ETA/B) and Angiotensin II type 1 (AT1) receptor in a Berlin cohort of ME/CFS patients (*n* = 116) by ELISA. The severity of disease, symptoms and autonomic dysfunction were assessed by questionnaires. Results: We found levels of most AABs significantly correlated with key symptoms of fatigue and muscle pain in patients with infection-triggered onset. The severity of cognitive impairment correlated with AT1-R- and ETA-R-AAB and severity of gastrointestinal symptoms with alpha1/2-AdR-AAB. In contrast, the patients with non-infection-triggered ME/CFS showed fewer and other correlations. Conclusion: Correlations of specific AAB against G-protein-coupled receptors (GPCR) with symptoms provide evidence for a role of these AAB or respective receptor pathways in disease pathomechanism.

## 1. Introduction

Myalgic Encephalomyelitis/Chronic Fatigue Syndrome (ME/CFS) is an acquired complex disease with cardinal symptoms of fatigue, post-exertional malaise (PEM), cognitive dysfunction and pain [1]. The estimated prevalence is up to 0.86%, with peaks in teenage years and middle age [2,3]. ME/CFS is triggered by an infection in the majority of patients [4]. Although the pathogenesis is still unknown, there is ample evidence of immune and autonomic dysregulation [5].

There is increasing evidence that vascular dysfunction and hypoperfusion play an important role in ME/CFS. A diminished oxygen supply in muscles upon exercise was shown in several studies in ME/CFS patients [6,7]. In line with this, metabolic changes in ME/CFS indicate hypoxia and ischemia [8]. Several studies showed a decrease in cerebral blood flow upon orthostatic challenge [9,10]. Thus, hypoperfusion, which is aggravated upon exertion, may cause mental and skeletal muscle fatigue that are hallmarks of ME/CFS [11].

For the regulation of blood flow, G-protein-coupled receptors (GPCR) for vasoactive hormones, such as catecholamines, acetylcholine, angiotensin II and endothelin 1, play an important role [12]. Regulatory autoantibodies (AAB) targeting GPCR are involved in the pathogenesis of many diseases. Anti-GPCR AAB bind to their corresponding receptors, which can result in both agonistic and antagonistic effects [13]. Among the first AAB to GPCR described were those to beta1 adrenergic receptor (AdR) in dilated cardiomyopathy and to angiotensin II type 1 receptor (AT1-R), mediating vasoconstriction as risk factors for renal transplant rejection [14,15]. AAB against GPCR has been found in many rheumatic diseases as well [16]. These AAB belong to a regulatory network, which is dysregulated in many diseases [17].

There is evidence that AdR and muscarinic acetylcholine receptors (M-AChR)-AAB play a role in ME/CFS, too. Tanaka et al. were the first to describe elevated M-AChR-AAB in ME/CFS and their association with muscle weakness and neurocognitive impairment [18]. In a previous study, we found elevated AAB against beta2-AdR as well as M3/M4-AChR in a subgroup of ME/CFS patients [19]. Bynke et al. were able to verify these findings detecting elevated AAB against beta1/2-AdR and M3/M4-AChR in serum but not in cerebrospinal fluid of ME/CFS patients [20]. Beta1/2-AdR-AAB levels in blood correlated with structural alterations in the brain related to pain modulation [21]. Recently, we found agonistic beta2-AdR-AAB in healthy controls and in ME/CFS patients, stimulating the beta2-AdR on immune cells and reporter cell lines. Importantly, this agonistic function was attenuated in ME/CFS [22]. When performing immunoadsorption to remove AAB from circulation, we observed short-term clinical improvement in most patients [23,24]. For ME/CFS patients receiving rituximab, we documented a sustained decline of pretreatment elevated beta2-AdR-AAB levels in clinical responders to rituximab treatment [19].

The aim of this study was to investigate correlations between levels of AAB binding to vasoregulative GPCR and the severity of clinical symptoms in ME/CFS. As AAB responses are frequently activated by infections, we distinguished between patients with and without infection triggered ME/CFS onset. In a recent study, we found an increased prevalence of the autoimmune associated single-nucleotide variants in CTLA4 and PTPN22 in ME/CFS patients with infectious disease onset only [25]. Catecholamines binding to alpha1/2-AdR on vascular smooth muscle cells cause vasoconstriction, while they mediate vasodilation via beta2-AdR. Angiotensin II binding to AT1-R and endothelin-1 to endothelin-1 type A and B receptor (ETA/B-R) both activate important vasoconstrictive pathways. These ligands are increased by physical exertion [12]. Protease-activated receptors (PAR) play a role in vasoregulation during inflammation. Activation of PAR-1 by thrombin was shown to induce vascular constriction [26,27]. PAR-2 activated by trypsin can mediate inflammatory cell adhesion to the endothelium [28]. Acetylcholine can mediate vasodilatation via M3-AChR dependent release of nitric oxide [29]. M4-AChR expression was described in the brain microvascular system [30]. We expected that if vasoregulative AAB levels play a role in the pathomechanism of ME/CFS, they should correlate with the severity of key symptoms and disability.

## 2. Materials and Methods

### 2.1. Patients

A total of 116 patients were diagnosed at the outpatient clinic for immunodeficiencies at the Institute for Medical Immunology at the Charité Universitätsmedizin Berlin between October 2016 and May 2017. Diagnosis of ME/CFS in all patients was based on the 2003 Canadian Consensus Criteria and exclusion of other medical or neurological diseases that may cause fatigue by a comprehensive clinical and laboratory evaluation [1]. All patients received a cardiopulmonary workup prior to referral. In case of suspected rheumatic, gastrointestinal or neurological disease, patients were referred to specialists before the diagnosis ME/CFS was given. The study was approved by the Ethics Committee of Charité Universitätsmedizin Berlin (EA4/090/10) in accordance with the 1964 Declaration of Helsinki and its later amendments. All patients gave informed consent.

### 2.2. Determination of Autoantibody Levels and Laboratory Blood Data

CellTrend GmbH, Luckenwalde, Germany, analyzed serum levels of AAB against alpha1-, alpha2-, beta1-, beta2-, beta3-AdR, M3- and M4-AChR; AT1-R, ETA-R and ETB-R; PAR1/2. Whole blood samples from each subject were allowed to clot at room temperature and then centrifuged at 2000× *g* for 15 min in a refrigerated centrifuge. The serum was purified and stored at −35 °C. The AAB were measured in serum samples using a sandwich ELISA kit (CellTrend GmbH, Luckenwalde, Germany). The microtiter 96-well polystyrene plates were coated with full-length receptor proteins. To maintain the conformational epitopes of the receptor, 1 mM calcium chloride was added to every buffer. Duplicate samples of a 1:100 serum dilution were incubated at 4 °C for 2 h. After washing steps, plates were incubated for 60 min with a 1:20,000 dilution of horseradish-peroxidase labelled goat anti-human IgG used for detection. In order to obtain a standard curve, the plates were incubated with test serum from a GPCR AAB-positive index patient. The ELISAs were validated according to the FDA’s “Guidance for industry: Bioanalytical method validation”. The concentration of serum IgG, IgA, IgM, IgE and IgG subclasses were determined at Charité diagnostics laboratory Labor Berlin GmbH.

### 2.3. Questionnaires for Symptom Scoring

The presence and severity of symptoms in patients with ME/CFS were assessed based on the 2003 Canadian Consensus Criteria [1,31]. Cardinal symptoms of fatigue, muscle pain, immune symptoms (mean of the 3 symptoms painful lymph nodes, sore throat and flu-like symptoms) and cognitive impairment (mean of the 3 symptoms memory disturbance, concentration ability and mental tiredness) were scored between 1 (no symptoms) and 10 (most severe symptoms) by the patients. Symptoms of autonomic dysfunction were assessed by the Composite Autonomic Symptom Score 31 (COMPASS 31) [32]. In addition, disability was examined using the Bell score focusing on the level of restriction in daily functioning [33] and fatigue using Chalder Fatigue Score [34]. Physical activities of daily life were assessed via the Short Form Health Survey 36 (SF-36) [35].

### 2.4. Statistical Analysis

Statistical data analyses were performed using IBM SPSS Statistics 22.0 (New York, NY, USA), GraphPad Prism 6.0 (San Diego, CA, USA) and R 4.0 (R Foundation for Statistical Computing, Vienna, Austria, http://www.R-project.org, accessed on 9 July 2021). All data were presented as median and interquartile range (IQR), mean and standard deviation (SD) or frequency (*n*) and percentage where appropriate. Comparisons of quantitative parameters between two groups were performed using the nonparametric Mann–Whitney test. Categorical parameters were compared between subgroups applying the Pearson’s χ_2_-test. Correlation analysis was performed using the nonparametric Spearman coefficient. Due to multiple testing, Benjamini–Hochberg (BH) correction was applied, aiming to control a false discovery rate of 5%. Adjusted *p*-values < 0.05 were considered to provide evidence for a statistically significant result.

## 3. Results

### 3.1. Cohort Characteristics

We analyzed a cohort of 116 ME/CFS patients for correlation of AAB levels with symptom severity. Patient characteristics are shown in Table 1. The median age was 43 years (IQR: 31–50), and the previous median duration of disease at the time of analysis was four years (IQR: 2–9). A total of 83 of the 116 patients (72%) were female, and 86 (74%) reported an infection-triggered onset of disease. Patients with infection-triggered onset were younger by a median difference of ten years (*p* = 0.005) and reported shorter disease duration (*p* = 0.022). There were no differences in symptom severity, Bell disability scale, SF-36 physical function and COMPASS 31-assessed autonomic dysfunction (Table 1) nor in AAB levels (Table 2) between these groups.

### 3.2. Correlation of AAB with Total IgG and Age

As we already observed in a previous study [19], most of the AAB levels showed a positive correlation with total IgG and IgG-subclasses, predominantly with IgG1 and IgG3 (Table S1). As the GPCR AAB belong to a regulatory network of AAB, their level may depend on total IgG levels. Further, we observed an inverse correlation with age for some AAB (Table S1), as well as between age and total IgG (whole cohort: r = −0.2526; *p* = 0.007, *n* = 114). Therefore, we calculated AAB/IgG ratios to correct for the effect of age (Table 2 and Table S2).

### 3.3. Correlation of AAB with Clinical Symptom Scores

**Figure 1:**
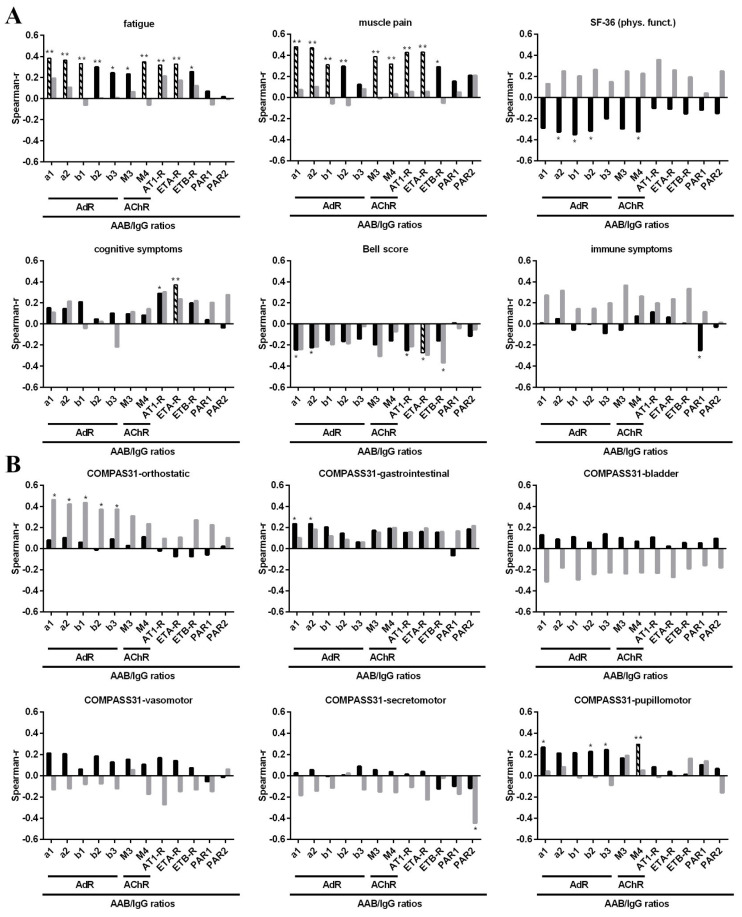
Correlations between symptom severity and AAB/IgG ratios. Correlation analysis of AAB/IgG ratios with the severity of (**A**) fatigue, muscle pain, cognitive and immune symptom scores, physical functioning (SF-36) and Bell disability score and (**B**) with COMPASS 31 subdomains. Spearman correlation coefficients (r) are shown for patients with infection-triggered onset (black bars) and patients without infection-triggered onset (grey bars). Significant correlations prior to BH-correction are marked with asterisks (* *p* < 0.05, ** *p* < 0.01), correlations that remained significant after BH-correction are indicated by black-and-white striped bars.

Levels of various AAB correlated with symptom severity (Table S3). Further, we observed a positive correlation of alpha1/2-AdR, M4-AChR and ETA-R with disease duration (Table S2). Minimizing the effect of age by using AAB/IgG ratios for correlation analyses led, in general, to higher correlation estimates (r) and more correlations reached a level of significance (Table S4). We analyzed patient cohorts according to the type of disease onset. As 74% of patients reported an infectious onset, this group was much larger than the non-infectious onset group. Correlations of symptom severity with AAB/IgG ratios stratified according to disease onset are shown as Spearman’s correlation coefficient values in Figure 1, and correlations of clinical symptoms with absolute AAB levels are shown in Figure S1. The most correlations were found in patients with infection-triggered onset only, while fewer and other correlations were found in those with non-infection-triggered onset (Tables S3 and S4).

In patients with infection-triggered onset (*n* = 86), severity of fatigue correlated positively with most AAB/IgG ratios, including those against alpha1/2-AdR, beta1/2/3-AdR, M3/4-AChR, AT1-R, ETA-R and ETB-R, but not PAR-1/2 (Figure 1A, black bars). Muscle pain severity showed similar correlations to fatigue, except for beta3-AdR-AAB/IgG. The SF-36 physical function showed a correlation pattern similar to fatigue and muscle pain with significant negative correlations (due to lower scores indicating more severe impairment) with alpha2-AdR-, beta1/2-AdR- and M4-ACh-AAB/IgG. In contrast, the severity of cognitive symptoms correlated positively with AT1-R- and ETA-R-AAB/IgG only. The severity of the Bell disability score showed a similar negative correlation with AT1-R- and ETA-R-, and with alpha1/2-AdR-AAB/IgG. For the immune score, only an inverse correlation with PAR1 was found. The Chalder Fatigue Score did not correlate with AAB/IgG (not shown). Scatter plots for significant correlations are shown in Figures S2–S5.

None of these significant correlations of fatigue and muscle pain were found in patients without infection-triggered onset (Figure 1A, grey bars). Correlations between most AAB/IgG and SF-36 physical function scores were even opposite to those of patients with infection-triggered onset. However, the correlation estimates (r) between AT1-R-AAB/IgG and cognitive symptoms and between AT1-R- and ETA-R-AAB/IgG and the Bell score were similar to those of patients with infection-triggered onset. As this subgroup was much smaller (*n* = 30), this may explain a lack of significance. Further, we found a significant negative correlation of ETB-R-AAB/IgG and the Bell score in this group only.

Interesting correlation patterns were also found for AAB/IgG and the three domains of orthostatic, gastrointestinal and pupillomotor function assessed by the COMPASS 31 questionnaire (Figure 1B). In patients with infection-triggered onset, the gastrointestinal symptoms correlated positively with alpha1/2-AdR- and the pupillomotor symptoms with alpha1-, beta2/3-AdR- and M4-AChR-AAB/IgG ratios. In contrast, the non-infection-triggered onset group showed strong correlations of alpha1/2-AdR- and beta1/2/3-AdR-AAB/IgG with orthostatic symptoms and an inverse correlation of PAR2-AAB/IgG with secretomotor symptoms, which are absent in the other subgroup. We did not observe any of these correlations with total IgG (not shown). Patients without infection-triggered onset had a significantly longer disease duration prior to these analyses (Table 1). As we observed higher alpha1/2-AdR, M4-AChR and ETA-R-AAB/IgG ratios to be associated with longer disease duration (Table S2), this may have an impact on the correlations of alpha1/2-AdR/IgG with orthostatic symptoms.

In patients with infection-triggered onset, most of the AAB/IgG ratio correlations with fatigue, muscle pain and cognitive symptoms, as well as Bell score with ETA-R-AAB/IgG, remained significant after BH-correction (Table 3 and Figure 1). In addition, the association of pupillomotor symptoms with M4-AChR-AAB/IgG remained significant. After BH correction, none of the correlations observed in patients without infection-triggered onset remained significant.

## 4. Discussion

There is increasing evidence for a role of vascular dysfunction in ME/CFS that shows associations with key symptoms [11]. In this study, we found several remarkable correlations of vasoregulative AAB with clinical symptoms in ME/CFS. The dependence between the measured biologic gradient of AAB and the severity of symptoms suggests a causal pathomechanistic connection.

Due to a correlation of natural regulative AAB with total IgG [19] and dependence of IgG levels on age [36], AAB/IgG ratios were used in our analyses in order to correct for the influence of age. Using the AAB/IgG ratios instead of absolute AAB levels revealed stronger and more correlations, and more *p*-values reached a level of significance.

In line with our hypothesis of a role of vasoactive AAB in ME/CFS, we found that levels of alpha1/2- and beta1/2/3-AdR-, M3/4-AChR-, and AT1-R-, ETA/B-R-AAB/IgG ratios all significantly correlate with the severity of fatigue and, with the exception of beta3-AdR-AAB/IgG, with muscle pain. The same AAB alpha2-AdR-, beta1/2-AdR- and M4-AChR- (but not AT1-R-, ETA/B-R-) correlated with SF-36 physical function. Tanaka et al. already described the levels of M-AChR-AAB (without data on the M subtype) in ME/CFS to be associated with muscle weakness [18]. Bynke et al. could not show an association of AAB against beta1/2-AdR and M3/M4-AChR with various health-related questionnaires, but their cohort was rather small, and key symptoms including fatigue, muscle pain, cognitive and autonomous symptoms were not separately assessed [20]. We found elevated AAB against beta2-AdR, as well as M3 and M4-AChR, in ME/CFS patients in our previous study [19]. In this study, the severity of symptoms was not determined. In patients with postural tachycardia syndrome (POTS), one study reported elevated levels of AAB against alpha1-AdR and M4-AChR to correlate with symptom severity [37], while another demonstrated elevated levels of AAB against beta1-AdR- and beta2-AdR to correlate with symptom severity [38].

We observed a distinct pattern for cognitive impairment, which was associated with ETA-R and AT1-R-AAB. Of interest, ETA/B-R-, AT1-R- and further alpha1/2-AdR AAB/IgG correlated with the severity of Bell disability score, too, capturing exertion induced symptoms and ability to work. ETA-R-, AT1-R- and alpha1/2-AdR all activate strong vasoconstrictor pathways stimulated by physical exertion [12]. Enhanced levels of AT1-R-AAB are a well-established risk factor for renal transplant rejection [14]. In hypertension, elevated AT1-R-AAB and alpha1-AdR-AAB have been described suggesting an agonistic effect on their receptors [39]. Furthermore, AT1-R-AAB were associated with vascular aging and arterial stiffness [40,41]. The role of ETA-R-AAB was described in autoimmune-related pulmonary arterial hypertension in both systemic lupus erythematosus and systemic sclerosis [42,43]. Our concept of higher ETA-R and AT1-R AAB/IgG to correlate with cognitive impairment due to vasoconstriction is in line with the recent studies by van Campen et al., showing both cerebral hypoperfusion and a decline in cognitive function in ME/CFS upon orthostatic stress [9,10].

Interesting correlation patterns were found for AAB/IgG ratios and gastrointestinal and pupillomotor function in the infection-triggered onset group as well. The gastrointestinal symptoms correlated with alpha1/2-AdR-ABB/IgG. This finding is in line with a study showing that colorectal motility is mediated by alpha1-AdR [44]. Pupillomotor symptoms correlated with alpha1-AdR-, beta2/3-AdR- and M4-AChR-ABB/IgG. Upon BH correction, the association of pupillomotor symptoms with M4-AChR-ABB/IgG remained significant. M4-AChR expression was described in the brain microvascular system and corneal endothelium [30,45].

Remarkably, we found no significant correlations of AAB/IgG against AdR, AChR and AT1-R/ET-R with fatigue, muscle pain and of AdR- and AChR-ABB/IgG with SF-36 physical function in patients without an infection-triggered onset of disease. However, similar estimates for correlations of AAB/IgG ratios to AT1-R/ETA-R and alpha1/2-AdR with cognition and Bell Score were found, which were not significant, likely related to the three-fold lower number of patients in this group. Only in patients with non-infectious disease onset significant correlations of symptoms of orthostatic intolerance with all AdR-AAB/IgG were seen. Further, an inverse correlation of PAR2-AAB/IgG with secretomotor symptoms was found. PAR2 activated by trypsin was shown to mediate salivary secretion [46].

GPCR AAB are different from classical autoantibodies that frequently activate, complement and can mediate inflammation and destruction [47]. No cytotoxic effect or complement activation of GPCR AAB has been reported so far. GPCR AAB specifically bind to their corresponding receptors, which can have functional consequences. Both stimulating agonistic and inhibiting antagonistic effects were described [13,15,48]. Considering an agonistic function, several associations of AAB with symptoms that we found are plausible. Elevated levels of agonistic AT1-R/ETA-R AAB could well explain the association with more cognitive dysfunction due to their effect on vasoconstriction described in several other diseases [49]. In a similar manner, enhanced PAR2 activity could explain fewer secretomotor symptoms and enhanced alpha1-AdR activity more gastrointestinal symptoms [44]. The inverse correlation of immune score with PAR1-AAB/IgG could be explained by lower levels of PAR1-AAB, resulting in less vascular constriction [26,27]. The associations of elevated levels of both alpha- and beta-AdR-AAB with more severe fatigue and muscle pain in post-infectious ME/CFS could point to overactivity of vasoconstrictive alpha-AdR-AAB or an impaired function of vasodilatative beta2-AdR-AAB. Previously, we found an impaired agonistic beta2-AdR-AAB function in immune and reporter cell line assays in ME/CFS patients with higher AAB levels [22]. AAB against beta1-AdR were shown to impair both beta1-AdR- and beta3-AdR-mediated vasorelaxation in rats [50]. The AdR dysfunction may specifically play a role upon exertion with enhanced release of epinephrine and norepinephrine, resulting in enhanced vasoconstriction and hypoperfusion with consecutive fatigue and muscle pain. Autoimmune mechanisms are likely in post-infectious ME/CFS [25]. As all these AAB are natural regulatory, AAB dysfunction may evolve during infection by bystander activation and somatic hypermutation resulting in AAB with a stronger or altered antigen binding to GPCR or in epitope spreading. With respect to AdR such a scenario would be in line with patients frequently reporting that an infection in a stressful situation, presumably going along with a stress-induced activation of AdR, triggered the disease onset.

The most discrepant patterns we observed between the patient subgroups are the correlations of all AdR-AAB with orthostatic dysfunction in non-infection-triggered disease but with fatigue, muscle pain and SF-36 in post-infectious ME/CFS. Since absolute AAB levels, as well as AAB/IgG ratios, did not differ between the two patient subgroups, this implicates that not merely the AAB level but rather the function of the AAB or of the receptors are different in these patient subgroups. To further follow this hypothesis, in patients with non-infection-triggered onset, the function of AdR-AAB responses may not be altered, but correlations here could reflect an adaptive response. For example, patients with connective tissue diseases, such as Ehlers Danlos syndrome (EDS), are at higher risk to develop ME/CFS. Here the vasculature is more elastic, leading to lower systolic and diastolic blood pressure, tachycardia and often POTS. Patients have an autonomic dysfunction with gastrointestinal problems and disturbed bladder function as well. There is evidence that patients with EDS have heightened vasoconstriction due to adrenergic hyper-responsiveness [51]. It is tempting to speculate that in these patients, elevated AdR-AAB are reflecting this compensatory overactivity of the adrenergic system. In line with this concept, in patients with POTS, elevated levels of AAB against alpha1-AdR and M4-AChR correlated with symptom severity [37].

A limitation of this study is that several correlations were no longer evident after correction for multiple testing due to the many parameters analyzed in our study. We provided both the corrected and the uncorrected correlations in order to address a possible unnecessary rejection of true findings upon adjustment for multiple testing [52]. The interpretation of the findings in the cohort without infection-triggered onset is based on a smaller number of patients and some non-significant findings. We did not increase the number of patients with non-infection triggered ME/CFS because we did not want to add a patient group diagnosed and analyzed at a later time point. In addition, disease onset is self-reported, and some patients may be wrongly classified. The symptom severity is self-reported and a subjective measure, leading to a wide distribution. As sleep disturbances are a key symptom in ME/CFS too, and sleep is associated with the parasympathetic system, a sleep score should be assessed in further studies. Further, we did not analyze a healthy control cohort in this study. In previous and ongoing unpublished studies, we constantly found that a subgroup of approximately one-third of ME/CFS patients has higher AAB against beta2-AdR as well as M3/M4-AChR compared to healthy controls [19,20]. Findings from our recent functional study suggest that the agonistic function of beta2-AdR AAB may be attenuated in ME/CFS patients, too, despite normal AAB levels [22].

In conclusion, our study provides evidence that AAB and/or the receptor pathways of AdR, AChR as well as AT1-R and ET-R play a role in ME/CFS due to the association with symptom severity. Thus, it is conceivable that various symptoms of ME/CFS, including fatigue, muscle pain, cognitive impairment and autonomic dysregulation, could be mediated or aggravated by these AAB. Further studies are required to decipher the mechanism and binding specificity of these GPCR-AAB, and their effect of on vascular function in ME/CFS, and how this may be translated into therapeutic concepts. In the case of dysfunctional AAB, therapies targeting AAB, such as immunoadsorption or rituximab, would be warranted and were shown to be effective in a subset of ME/CFS patients (reviewed in [5]). Further specific targeting of dysfunctional or regulative AAB may be developed as treatment strategies in ME/CFS.

## 5. Patents

CellTrend GmbH holds a patent for the use of beta-adrenergic receptor antibodies in the diagnosis of CFS.

## Figures and Tables

**Table 1:** Clinical characteristics. Asterisks mark significant differences between groups (Mann–Whitney test, * *p* < 0.05, ** *p* < 0.01).

	Whole Cohort (*n* = 116, Median with IQR)	w/Infection-Triggered Onset (*n* = 86, Median with IQR)	w/o Infection-Triggered Onset (*n* = 30, Median with IQR)	Inf. vs. Non-Inf.
Age	42.5a (31–50)	39a (31–47)	49a (40–54)	*p*: 0.005 **
Disease duration	4a (2–9)	3a (1–8)	6.50a (2.00–14.25)	*p*: 0.022 *
Sex (f/m)	83/33 (72%/28%)	64/22 (74%/26%)	19/11 (63%/37%)	*p*: 0.247
Fatigue	8 (7–9)	8 (7–9)	8.50 (8–10)	*p*: 0.113
Cognitive-score	7 (5.67–8.00)	7.21 (5.92–8.00)	6.84 (5.67–7.96)	*p*: 0.351
Muscle pain	7 (5–8)	7 (5–8)	8 (6.00–8.38)	*p*: 0.187
Immune-score	5.33 (4.00–6.67)	5.66 (4.17–7.00)	5.17 (3.67–5.96)	*p*: 0.226
Bell-Score	30 (30–40)	30 (30–40)	30 (30–40)	*p*: 0.560
Chalder-Fatigue Score	27 (25–30)	28 (25.88–30)	26 (24–30)	*p*: 0.130
SF-36 Score physical function	45 (20–55)	45 (18.75–61.25)	40 (30–50)	*p*: 0.834
COMPASS 31 total score	45.70 (35.18–55.42)	45.47 (34.36–55.34)	46.37 (39.28–56.13)	*p*: 0.687
COMPASS 31 orthostatic score	28 (20–32)	28 (20–32)	28 (20–32)	*p*: 0.954
COMPASS 31 vasomotoric score	0 (0–3)	0 (0–3)	0 (0–3)	*p*: 0.646
COMPASS 31 secretomotoric score	6.42 (3.75–8.56)	6.42 (2.14–8.56)	6.42 (4.28–8.56)	*p*: 0.294
COMPASS 31 gastrointestinal score	8.90 (6.90–12.46)	8.90 (6.23–12.46)	8.90 (7.12–12.02)	*p*: 0.932
COMPASS 31 bladder score	1.10 (0–2.20)	1.10 (0–2.20)	0 (0–2.20)	*p*: 0.369
COMPASS 31 pupillomotoric score	2.40 (1.43–3.00)	2.40 (1.50–3.00)	2.40 (1.20–3.00)	*p*: 0.772

**Table 2:** AAB levels and AAB/IgG-ratios. Differences between groups analyzed using Mann–Whitney test.

	Whole Cohort (*n* = 116, Median with IQR)	w/Infection- Triggered Onset (*n* = 86, Median with IQR)	w/o Infection- Triggered Onset (*n* = 30, Median with IQR)	Inf. vs. Non-Inf.
alpha1-AdR-AAB	8.71 U/l (7.24–11.65)	8.66 U/l (7.30–11.86)	8.83 U/l (6.85–10.02)	*p*: 0.400
alpha2-AdR-AAB	7.36 U/l (6.06–9.11)	7.37 U/l (6.05–9.65)	7.34 U/l (6.07–8.97)	*p*: 0.709
beta1-AdR-AAB	10.30 U/l (7.86–15.20)	9.88 U/l (7.61–16.20)	10.67 U/l (8.48–13.44)	*p*: 0.902
beta2-AdR-AAB	6.74 U/l (4.76–11.26)	6.74 U/l (4.75–11.55)	6.59 U/l (4.67–10.37)	*p*: 0.622
beta3-AdR-AAB	8.93 U/l (6.10–13.31)	9.45 U/l (6.29–13.68)	8.70 U/l (5.72–13.20)	*p*: 0.824
M3-AChR-AAB	4.74 U/l (3.41–6.10)	4.77 U/l (3.44–7.01)	4.45 U/l (3.37–5.62)	*p*: 0.293
M4-AChR-AAB	6.50 U/l (5.16–8.33)	6.50 U/l (5.20–9.11)	6.59 U/l (5.08–7.98)	*p*: 0.660
AT1-R-AAB	11.28 U/l (8.52–16.38)	11.62 U/l (8.50–17.05)	10.49 U/l (8.68–16.13)	*p*: 0.474
ETA-R-AAB	9.03 U/l (7.65–12.45)	8.98 U/l (7.60–12.79)	9.77 U/l (7.87–11.46)	*p*: 0.774
ETB-R-AAB	13.05 U/l (10.00–19.67)	13.05 U/l (10.03–19.87)	13.06 U/l (9.71–17.48)	*p*: 0.750
PAR1-AAB	4.52 U/l (3.14–5.96)	4.76 U/l (3.26–6.31)	3.49 U/l (3.07–4.91)	*p*: 0.102
PAR2-AAB	12.80 U/l (9.33–21.48)	12.12 U/l (8.46–22.08)	14.63 U/l (10.68–18.50)	*p*: 0.535
alpha1-AdR-AAB/IgG	0.90 U/g (0.78–1.20)	0.90 U/g (0.78–1.21)	0.90 U/g (0.77–1.13)	*p*: 0.626
alpha2-AdR-AAB/IgG	0.75 U/g (0.64–0.98)	0.74 U/g (0.63–0.99)	0.78 U/g (0.65–0.98)	*p*: 0.969
beta1-AR-AAB/IgG	1.06 U/g (0.82–1.54)	1.02 U/g (0.80–1.47)	1.14 U/g (0.86–1.57)	*p*: 0.595
beta2-AdR-AAB/IgG	0.71 U/g (0.49–1.12)	0.70 U/g (0.49–1.11)	0.78 U/g (0.44–1.15)	*p*: 0.897
beta3-AdR-AAB/IgG	0.88 U/g (0.66–1.31)	0.88 U/g (0.66–1.28)	0.89 U/g (0.67–1.49)	*p*: 0.989
M3-AChR-AAB/IgG	0.48 U/g (0.37–0.64)	0.48 U/g (0.37–0.66)	0.46 U/g (0.33–0.62)	*p*: 0.479
M4-AChR-AAB/IgG	0.69 U/g (0.51–0.87)	0.69 U/g (0.51–0.88)	0.68 U/g (0.52–0.88)	*p*: 0.984
AT1-R-AAB/IgG	1.16 U/g (0.92–1.73)	1.19 U/g (0.93–1.77)	1.14 U/g (0.85–1.47)	*p*: 0.414
ETA-R-AAB/IgG	0.95 U/g (0.77–1.29)	0.93 U/g (0.76–1.34)	1.03 U/g (0.81–1.18)	*p*: 0.812
ETB-R-AAB/IgG	1.29 U/g (1.02–1.93)	1.31 U/g (1.02–2.00)	1.29 U/g (1.00–1.82)	*p*: 0.707
PAR1-AAB/IgG	0.45 U/g (0.35–0.59)	0.45 U/g (0.36–0.66)	0.36 U/g (0.31–0.52)	*p*: 0.067
PAR2-AAB/IgG	1.37 U/g (0.96–1.99)	1.36 U/g (0.92–1.98)	1.55 U/g (1.15–2.16)	*p*: 0.398
total IgG	9.73 g/l (8.39–11.10)	9.79 g/l (8.41–11.09)	9.63 g/l (8.36–11.51)	*p*: 0.969

**Table 3:** Significant clinical correlations with AAB/IgG ratios after BH-correction in patients with infection-triggered onset (Spearman correlation coefficient and Benjamini–Hochberg corrected *p*-value; significant correlations marked with asterisks: * *p* < 0.05, ** *p* < 0.01).

	alpha1-AdR-AAB/IgG	alpha2-AdR-AAB/IgG	beta1-AdR-AAB /IgG	M3-AChR- AAB /IgG	M4-AChR- AAB/IgG	AT1-R- AAB/IgG	ETA-R- AAB/IgG
Fatigue	r: 0.383 *p*: 0.004 **	r: 0.363 *p*: 0.009 **	r: 0.331 *p*: 0.045 *	r: 0.234 *p*: 0.280	r: 0.349 *p*: 0.028 *	r: 0.317 *p*: 0.035 *	r: 0.328 *p*: 0.017 *
Muscle pain	r: 0.482 *p*: <0.001 **	r: 0.471 *p*: <0.001 **	r: 0.310 *p*: 0.045 *	r: 0.386 *p*: 0.008 **	r: 0.319 *p*: 0.035 *	r: 0.427 *p*: 0.002 **	r: 0.429 *p*: 0.001 **
Cognitive score	r: 0.152 *p*: 0.303	r: 0.144 *p*: 0.306	r: 0.209 *p*: 0.132	r: 0.095 *p*: 0.583	*p*: 0.084 *p*: 0.589	r: 0.290 *p*: 0.051	r: 0.371 *p*: 0.007 **
Bell Score	r: −0.244 *p*: 0.099	r: −0.223 *p*: 0.105	r: −0.154 *p*: 0.270	r: −0.196 *p*: 0.280	r: −0160 *p*: 0.308	r: −0.250 *p*: 0.083	r: −0.273 *p*: 0.045 *
COMPASS 31 pupillomotoric score	r: 0.268 *p*: 0.082	r: 0.212 *p*: 0.123	r: 0.215 *p*: 0.132	r: 0.166 *p*: 0.298	r: 0.294 *p*: 0.042 *	r: 0.084 *p*: 0.590	r: 0.039 *p*: 0.791

## Data Availability

The data presented in this study are available on reasonable request from the corresponding author.

## Supplementary Materials

The following are available online at https://www.mdpi.com/article/10.3390/jcm10163675/s1, Table S1: AAB para-clinical correlations, Table S2: AAB/IgG ratio para-clinical correlations, Table S3: AAB clinical correlations of AdR-, AChR- and other AAB; onset-stratified, Table S4: Clinical correlations of AdR-, AChR- and other AAB/IgG ratio; onset-stratified, Figure S1: Correlations between symptom severity and AAB, Figure S2: Correlations with fatigue in patients with infection-triggered onset, Figure S3: Correlations with muscle pain, cognitive and immune symptoms in patients with infection-triggered onset, Figure S4: Correlations with Bell score, physical functioning and symptoms of autonomic dysfunction in patients with infection-triggered onset, Figure S5: Correlations in patients without infection-triggered onset.
