# Supplementary material for: Autoantibodies to Vasoregulative G-Protein-Coupled Receptors Correlate with Symptom Severity, Autonomic Dysfunction and Disability in Myalgic Encephalomyelitis/Chronic Fatigue Syndrome
Source: J Clin Med. 2021 Aug 19;10(16):3675. doi: 10.3390/jcm10163675 (PMC8397061; doi:10.3390/jcm10163675)

## Slide 1
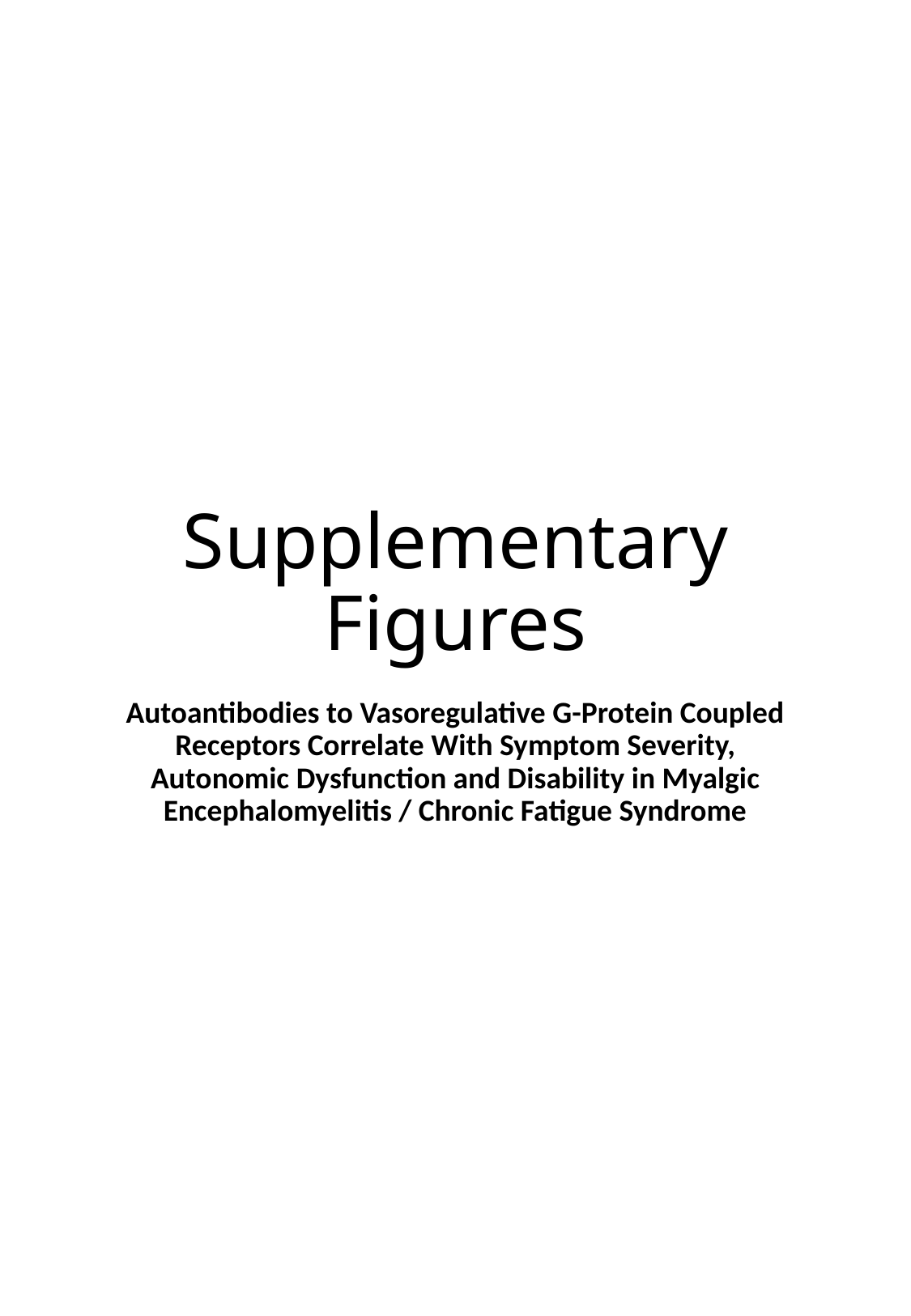

# Supplementary Figures
Autoantibodies to Vasoregulative G-Protein Coupled Receptors Correlate With Symptom Severity, Autonomic Dysfunction and Disability in Myalgic Encephalomyelitis / Chronic Fatigue Syndrome

## Slide 2
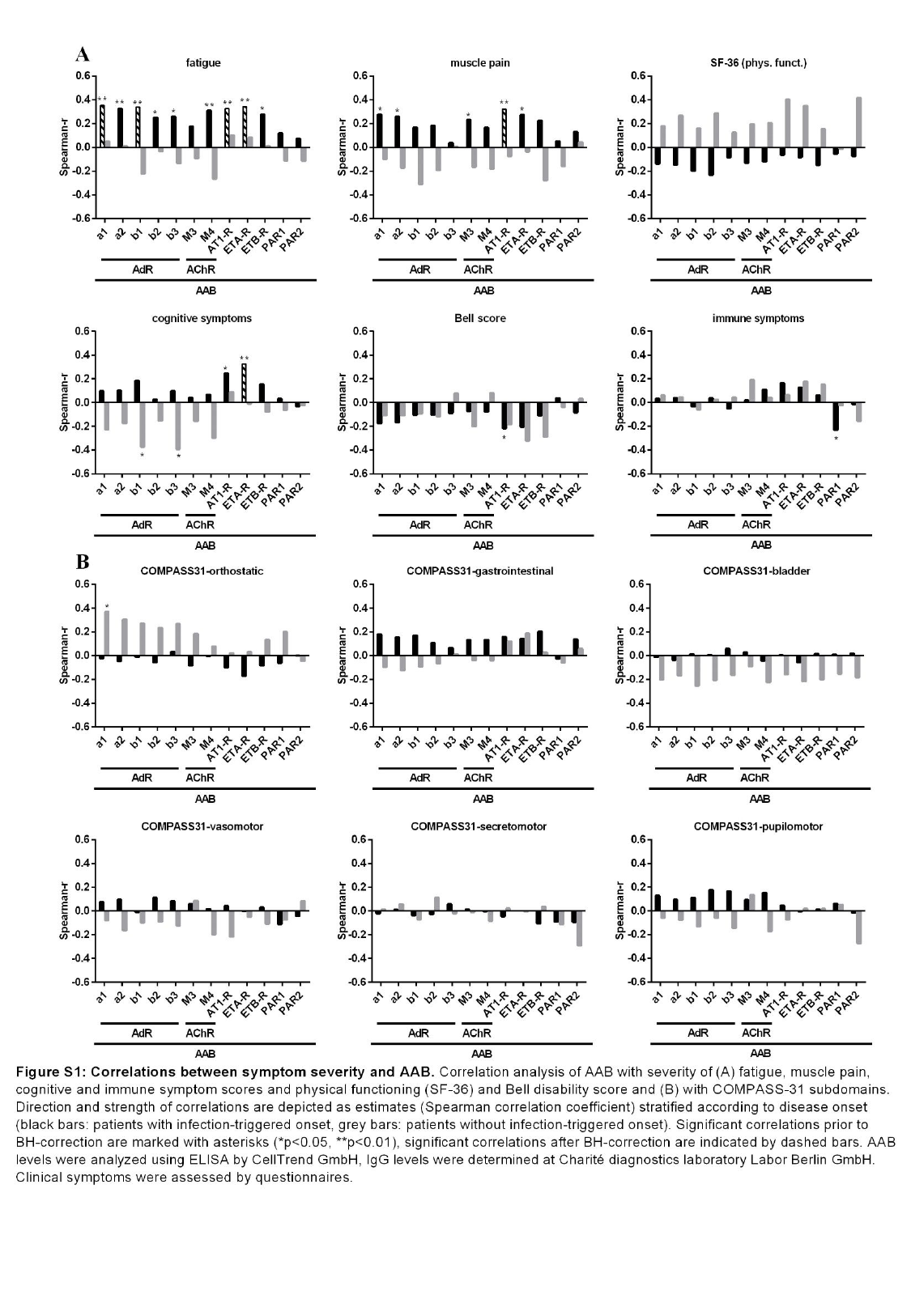

## Slide 3
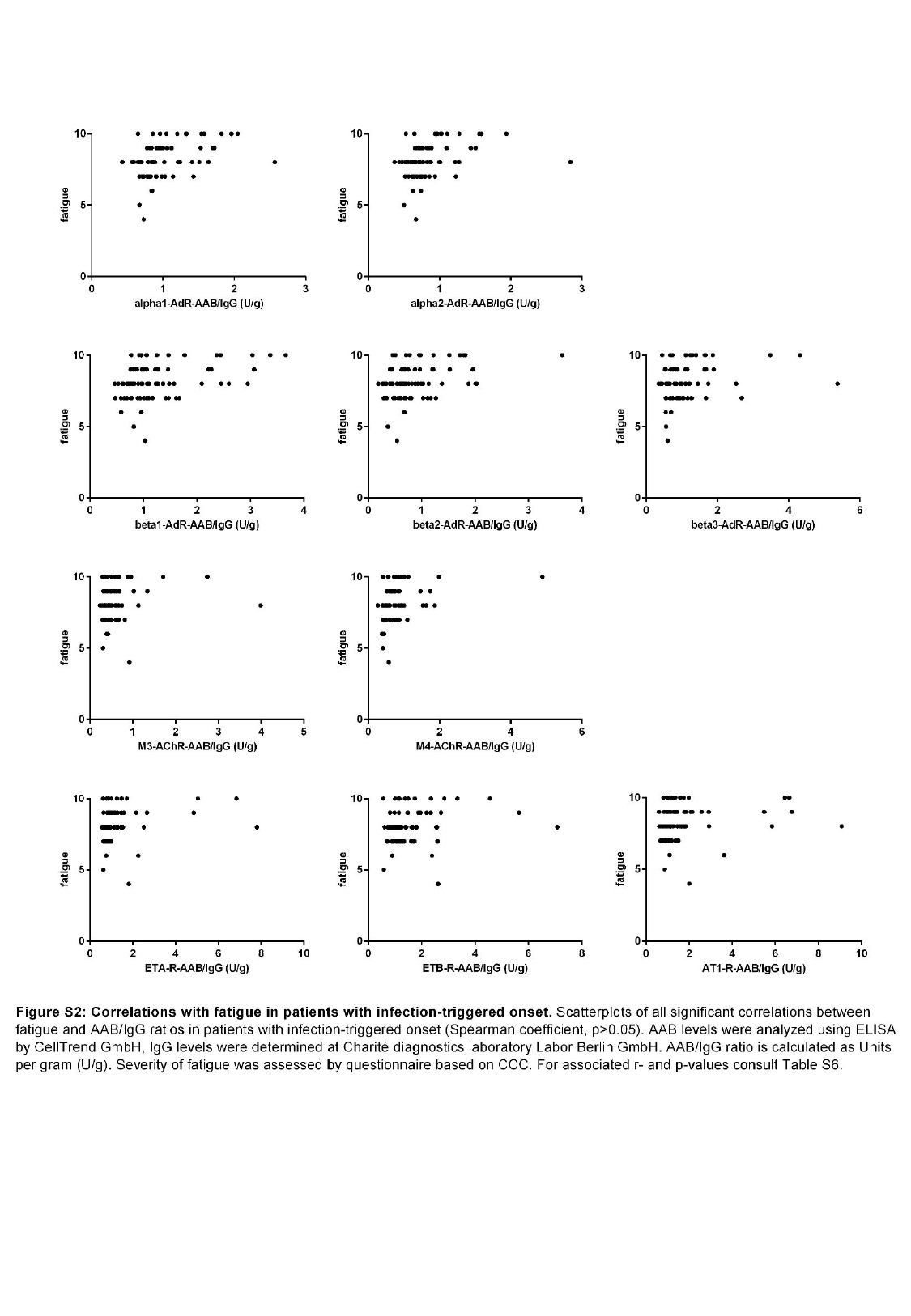

## Slide 4
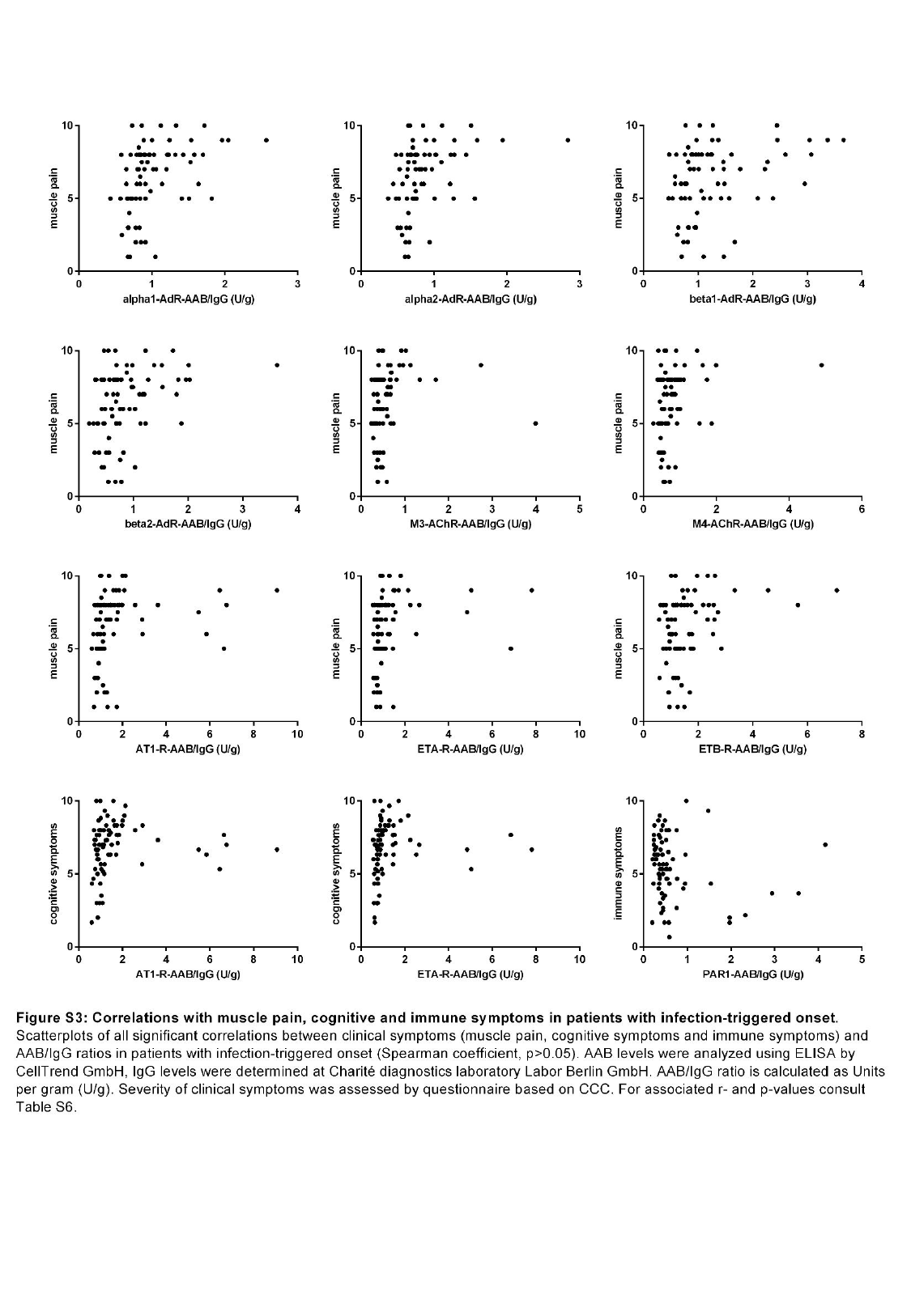

## Slide 5
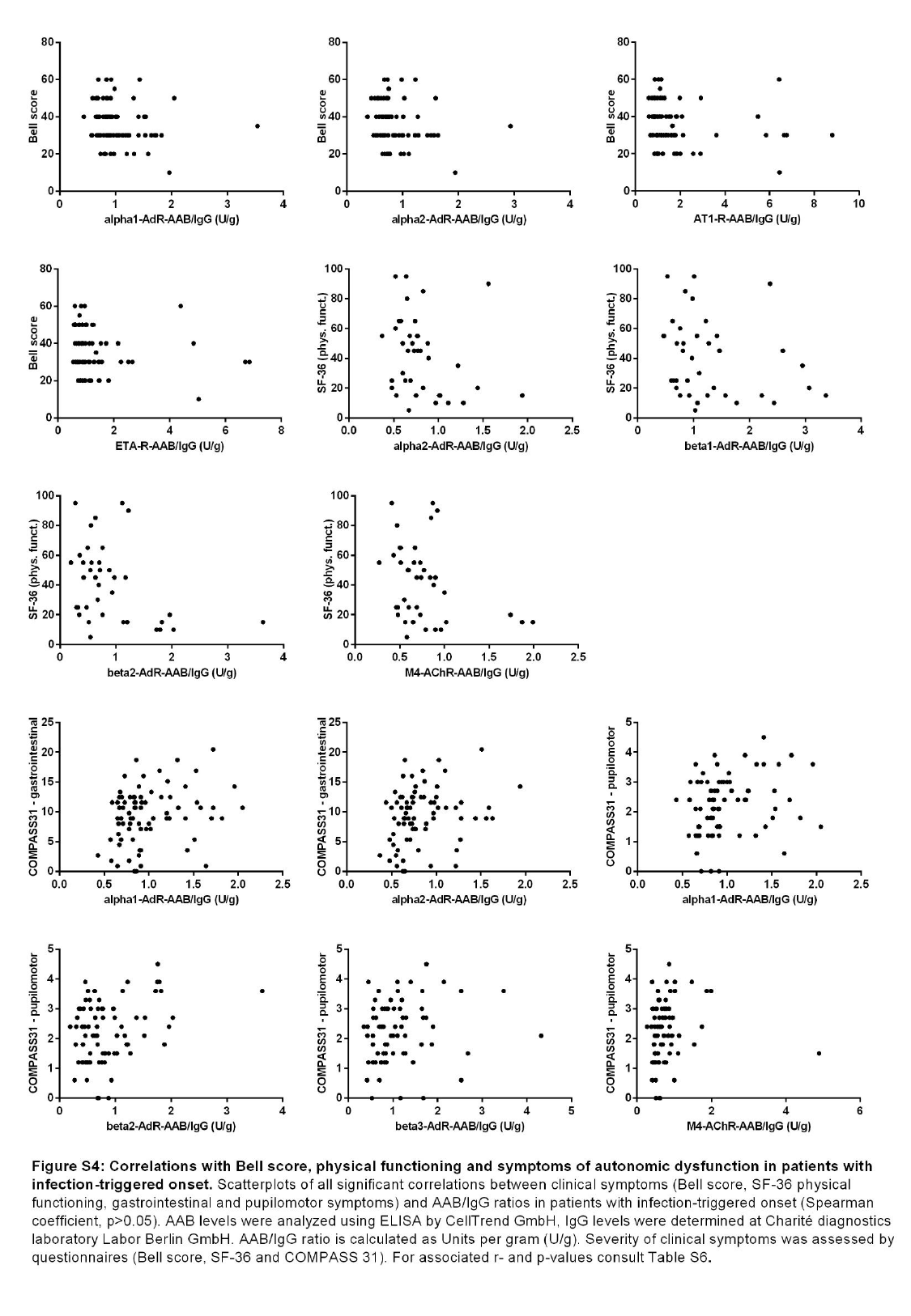

## Slide 6
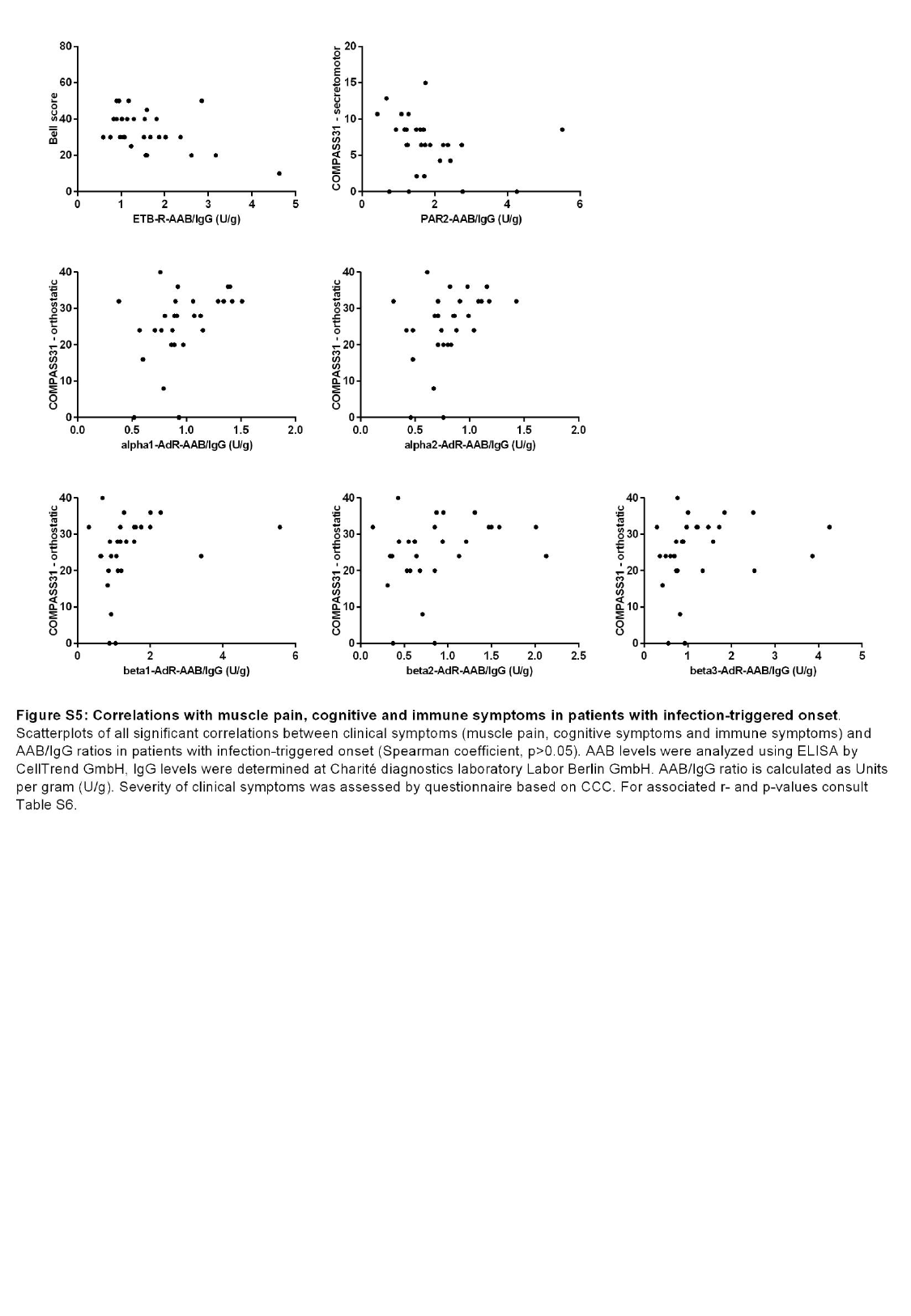

Supplement: Supplementary file 1 [file jcm-10-03675-s001.zip › Supplementary_Figures_JCM_.pptx]
